# Comparing Methodologies for Stomatal Analyses in the Context of Elevated Modern CO_2_

**DOI:** 10.3390/life14010078

**Published:** 2024-01-02

**Authors:** Rebekah A. Stein, Nathan D. Sheldon, Selena Y. Smith

**Affiliations:** 1 Department of Chemistry & Physical Sciences, Quinnipiac University, Hamden, CT 06518, USA; 2 Department of Earth and Environmental Sciences, University of Michigan, Ann Arbor, MI 48109, USA

**Keywords:** stomata, carbon dioxide, photosynthesis, paleobarometer, paleoclimate, atmosphere

## Abstract

Leaf stomata facilitate the exchange of water and CO_2_ during photosynthetic gas exchange. The shape, size, and density of leaf pores have not been constant over geologic time, and each morphological trait has potentially been impacted by changing environmental and climatic conditions, especially by changes in the concentration of atmospheric carbon dioxide. As such, stomatal parameters have been used in simple regressions to reconstruct ancient carbon dioxide, as well as incorporated into more complex gas-exchange models that also leverage plant carbon isotope ecology. Most of these proxy relationships are measured on chemically cleared leaves, although newer techniques such as creating stomatal impressions are being increasingly employed. Additionally, many of the proxy relationships use angiosperms with broad leaves, which have been increasingly abundant in the last 130 million years but are absent from the fossil record before this. We focus on the methodology to define stomatal parameters for paleo-CO_2_ studies using two separate methodologies (one corrosive, one non-destructive) to prepare leaves on both scale- and broad-leaves collected from herbaria with known global atmospheric CO_2_ levels. We find that the corrosive and non-corrosive methodologies give similar values for stomatal density, but that measurements of stomatal sizes, particularly guard cell width (GCW), for the two methodologies are not comparable. Using those measurements to reconstruct CO_2_ via the gas exchange model, we found that reconstructed CO_2_ based on stomatal impressions (due to inaccurate measurements in GCW) far exceeded measured CO_2_ for modern plants. This bias was observed in both coniferous (scale-shaped) and angiosperm (broad) leaves. Thus, we advise that applications of gas exchange models use cleared leaves rather than impressions.

## 1. Introduction

The inverse relationship between the quantity of stomata on leaf surfaces and the concentration of atmospheric carbon dioxide ([CO_2_]) has been documented and established as a tool for reconstructing paleo-CO_2_ [1,2,3,4]. This inverse relationship is driven by the resource cost of stomata, as though there is active stomatal closure, these pores can be "leaky," and a higher density of the cells can result in higher transpiration rates [5,6,7]. Thus, in times of high CO_2_, leaves can reduce the number of stomata (i.e., lower the density of stomata on the leaf surface) and still assimilate sufficient CO_2_ [8,9,10,11]. However, other environmental factors (e.g., water stress) can impact stomatal density, and other stomatal features, such as their size (i.e., width, length, area, and shape), are not constant across plant groups and may be influenced by environmental factors as well [12]. As such, mechanistic models have been introduced to incorporate additional stomatal variables [13,14].

The inverse relationship between stomatal density and [CO_2_] has been tested on fossil plants and within highly controlled growth chamber experiments [13,15,16], but it is less clear how quickly plants respond to changes in atmospheric CO_2_ in the natural world [17]. For example, many plants show little or no change in C isotope assimilation over the period of Industrialization [18,19,20,21] in spite of increasing [CO_2_]. Due to anthropogenic climate change, it is imperative that we understand how adaptive plants are to rising CO_2_, such that we can quantify the biosphere’s potential as a carbon sink [22,23,24] and predict crop yields [25,26,27]. Excessively slow responses of stomatal parameters can be problematic because, in addition to being inlets for CO_2_, these stomata are outlets for water, which could result in excessive water leakage and limit plant growth, especially in water-limited environments [28].

In addition to density, stomatal cell parameters such as guard cell length (GCL) and guard cell width (GCW) are controlled by environmental conditions in the short term and by evolution over long time scales. Previous work has shown that CO_2_ reconstruction proxies based on stomata are most accurate when considering stomata size as well [29]. Genome size is strongly predictive of GCL in angiosperms [29,30], suggesting there could be an evolutionary component to the stomatal response to stress; genome downsizing may have played a role in the evolution of angiosperms, facilitating higher densities of smaller stomata [31,32]. However, environmental factors also drive guard cell size. GCL in *Arabidopsis thaliana* also responded to atmospheric CO_2_, relative humidity, irradiance, UV radiation, and pathogens [33]. Specifically, relative humidity increases from 45 to 85% in controlled growth experiments caused an increase in 3% GCL. A controlled increase in CO_2_ from 380 to 3000 ppm and water availability from 10 mL d^−1^ to constant saturation both resulted in an increase in GCL of 6%; high irradiation increased GCL by 7%; and increased pathogens increased GCL by 10% [33]. Stomatal size and stomatal density may not be independent, as some workers report an inverse relationship between stomatal density and size [34,35,36,37,38]. Therefore, considering the full picture of the total surface area covered by stomata (stomatal size × stomatal density) can elucidate the total potential gas exchange capacity of a given leaf.

In this study, we collected a suite of modern and historical specimens from the families Cupressaceae (cypresses, junipers, and relatives) and Salicaceae (poplar, willows, and relatives) and measured stomatal parameters using two methodologies. One newer, non-toxic methodology (stomatal impression) uses tape and nitrocellulose dissolved in ethyl acetate (i.e., nail polish) to examine stomatal parameters [39,40]. The other methodology uses toxic, corrosive chromium trioxide (CrO_3_ [41,42]) or sodium hydroxide (NaOH [43]), which have been the conventional approaches since 1949. The goal of this work is to identify the strengths and weaknesses of each method in determining inputs for stomatal density-based and gas exchange model-based paleo-CO_2_ reconstructions by examining discrepancies in measurement sizes and outputs from the Franks et al. (2014) [13] model. This expands upon work done by Smith et al. (2014) [44] examining the difference between CO2-reconstructions based on macerations and impressions for stomatal frequency in *Ginkgo*. We compared the reconstructed [CO_2_] based on each method with known values measured at Mauna Loa Observatory, further helping us to identify the limitations of the impression compared with more typical leaf-clearing methodologies.

## 2. Materials and Methods

### 2.1. Specimen Sampling and Preparation

One hundred and seven total specimens from four species were sampled from herbarium (MICH) and field collections across North America. The focal genera, *Thuja* and *Populus*, are common temperate woody plants found throughout North America. There was no overlap in sites for the two species, but the ranges overlapped. *Thuja* sp. (n = 24), including *Thuja occidentalis* and *Thuja plicata*, and *Populus tremuloides* (n = 82) leaves were sampled from across field and herbarium collections. Molds of the leaf surfaces of intact, dried specimens were prepared using polyvinyl siloxane (dental putty; Affinis^®^ wash Light Body, Coltene, Altstätten, Switzerland). Clear nitrocellulose in ethyl acetate (i.e., nail polish) was applied to these dental putty molds, then stripped using packing tape and placed directly on a glass slide for microscope analysis, producing an impression of the surface of the leaf. A subset of *Thuja* leaves (n = 10) and *Populus* leaves (n = 20) was also cleared using a 20% CrO_3_ solution and a 5% NaOH solution, respectively, to emphasize morphological features such as stomata [45] for comparison. It was not possible to reuse the exact leaf surfaces for maceration and impressions, but the same leaves were used. Once cleared, specimens were rinsed and run through an ethanol dehydration series before being mounted on glass slides in glycerin or between cellulose acetate and cedarwood oil, respectively, for analysis. No cover slides were placed.

### 2.2. Microscopy

Photographs of the cuticular morphology of each leaf were taken on a NikonLV100 optical microscope in the Plant Evolution, Paleobotany, and Paleoecology Research Laboratory at the University of Michigan using Nikon Instrument’s NIS-Elements v.5.2 software. Approximately three images were taken per slide at either 200× or 400× magnification (Table S1). Following photography, stomatal parameters (density, GCL, and GCW) were measured in ImageJ (v1.54) software [46,47] in a field area of ~0.10 mm^2^, exceeding the minimum value required for density measurements [48]. Density was measured as number of stomata per area (mm^2^), then converted to m^2^ three times, and then the average was reported. We excluded areas where stomata were not present [13] to account for species-specific patterns of stomatal distribution. GCL and GCW were measured six times total in μm (two separate stomata per image, three images total) and converted into m and averaged [15].

### 2.3. C Isotope Measurements

Isotope measurements of each specimen were conducted in the Earth Systems Science Lab at the University of Michigan, Ann Arbor. Each leaf sample was run in duplicate with two International Atomic Energy Agency standards: IAEA-CH-6 (sucrose: −10.45‰) and IAEA-600 (caffeine: −27.77‰) in addition to internal laboratory standards (acetanilide: −26.58‰). See Stein et al. (2019; 2021) [20,21] for detailed isotope analysis approaches. Environmental variables, including δ^13^C_atm_ and [CO_2_] as measured at Mauna Loa Observatory (MLO [49]), mean annual precipitation, and mean annual temperature, were collected using PRISM Climate Group (2004) data [50].

### 2.4. Gas Exchange Model

Gas exchange calculations were conducted on both genera (*Thuja* and *Populus*) similarly, using different constants for species as discussed below. Isotope and stomatal parameter data were input into the Franks et al. (2014) model [13] (Equation (1)), where *c_a_* is [CO_2_], *A_n_* is total CO_2_ assimilation rate, *g_c_*_(*tot*)_ is total leaf conductance to CO_2_, and *C_i_*/*C_a_* is the ratio of internal to atmospheric CO_2_. To solve for *c_i_*/*c_a_*, we input known constants “*a*”: carbon isotope fractionation due to diffusion of CO_2_ in air (4.4‰), and “*b*”: fractionation associated with RuBisCO (30‰), as well as measured isotope values. *δ*^13^*C_atm_* values were measured at Mauna Loa Observatory [43], and *δ*^13^*C_leaf_* values were measured at University of Michigan.

(1) $$c_{a}=A_{n}/(g_{c\left(tot\right)}\;\left(1-\frac{C_{i}}{C_{a}}\right))$$

(2) $$\frac{c_{i}}{c_{a}}=\frac{[\frac{{(\delta }^{13}C_{atmosphere}-\delta^{13}C_{leaf})}{1+\left(\frac{\delta^{13}C_{leaf}}{1000}\right)}]-a}{b-a}$$

To calculate *g_c_*_(*tot*)_, a necessary parameter to reconstruct atmospheric CO_2_ (see Equation (1)), the leaf conductance model incorporates total possible conductance, which includes leaf boundary layer conductance to CO_2_, operational conductance, and mesophyll conductance [13]. Practically speaking, this requires a measurement to calculate *g_c_*_(*max*)_, or maximum stomatal conductance, which is dependent on both the number and size of stomata. The number of stomata, or SD (stomatal density), and stomatal size were measured in ImageJ. To estimate size (specifically, size for conductance capacity), total stomatal aperture (*a_max_*) and depth were used, which were determined with direct measurements of guard cell width, guard cell length, and stomatal pore length (see Equation (4), β is determined in Franks et al., 2014 [13]).

(3) $$g_{c(max)}=\frac{\frac{d}{v}\;SD\times a_{max}}{(1+\frac{\pi }{2}\sqrt{\frac{a_{max}}{\pi })}}$$

(4) $$a_{max}=\beta \;(\frac{\pi p^{2}}{4})$$

Combined, these equations create the full model in Equation (5), where *A_n_*, *b*, *a*, *d*, β, and *v* are constants as determined in Franks et al. (2014) [13] and by known kinetic and equilibrium carbon isotope fractionation in plants, as mentioned above [51]. Stomatal density (*SD*), *p*, *δ*^13^*C_atm_*, and *δ*^13^*C_leaf_* values are input based on our measurements.

(5) $$c_{a}=\frac{A_{n}\;}{\frac{\frac{d}{v}\;SD\times \beta \;(\frac{\pi p^{2}}{4})}{\left(1+\frac{\pi }{2}\sqrt{\frac{\beta \;(\frac{\pi p^{2}}{4})}{\pi }}\right)}\;\left(1-\frac{[\frac{{(\delta }^{13}C_{atmosphere}-\delta^{13}C_{leaf})}{1+\left(\frac{\delta^{13}C_{leaf}}{1000}\right)}]-a}{b-a}\right)}\;$$

This model uses measured stomatal density, GCL on both adaxial and abaxial surfaces, GCW on abaxial and adaxial surfaces, pore length, isotopic value of the leaves, isotopic value of the atmosphere, and [CO_2_]. As GCW increases and as GCL and stomatal density decrease, model-based estimates of CO_2_ increase. As *δ*^13^*C_leaf_* values become more positive, model-based estimates of CO_2_ increase. Additionally, the model uses prescribed boundary layer conductance to CO_2_, present-day *c_i_*/*c_a_* [13], and photosynthetic rate (*A*_0_), which we prescribe based on Franks et al. (2014) [13] supplemental methods (*c_i_*/*c_a_* = 0.62, *A*_0_ = 14.8 for angiosperms, *Populus tremuloides*; and *c_i_*/*c_a_* = 0.50, *A*_0_ = 7.9 for gymnosperms, genus *Thuja*). Inferring a starting *A*_0_ value increases a degree of uncertainty [13]. The standard error of 10% included in the mechanistic model addresses this, but nonetheless, it is worth noting that there are a number of assumptions made in choosing these values. We prescribed pore length (prescribed the code *s1* in the gas exchange model) as 0.33 for gymnosperms [13] and 0.30 for small angiosperm leaves.

## 3. Results

### 3.1. Method Comparisons

**Figure 1:**
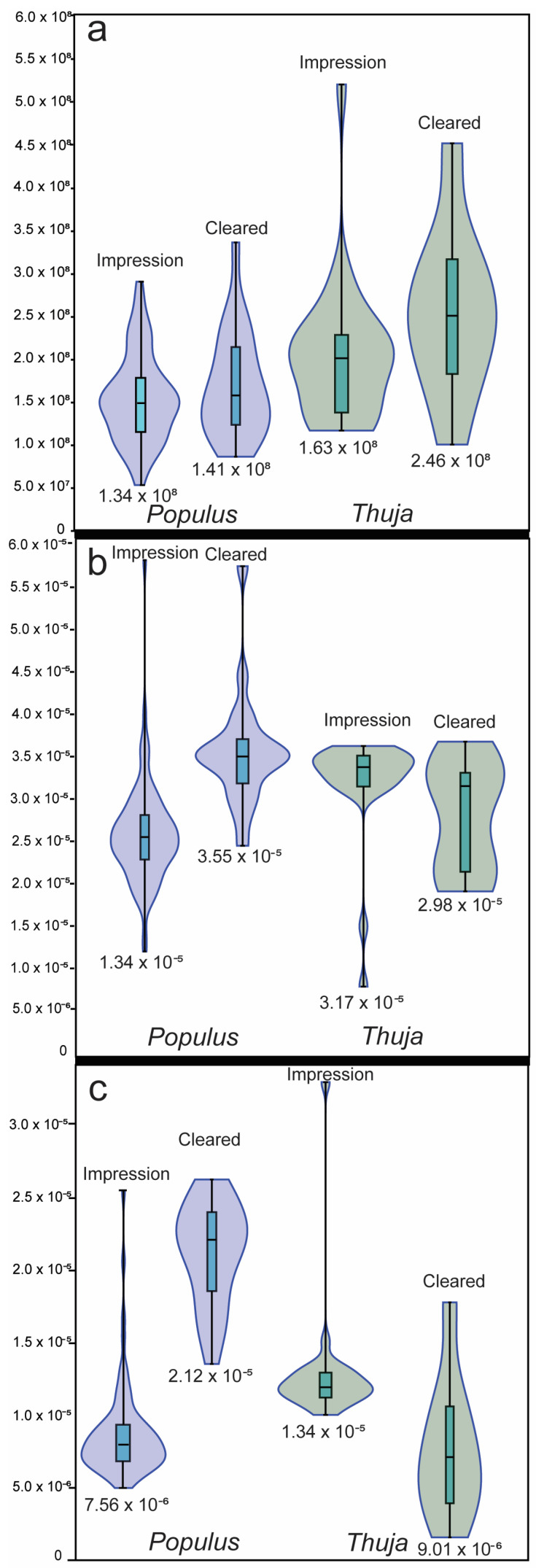
(**a**) violin plots of stomatal density for all species in impressions and cleared leaves (in stomata m^−2^), with average labeled. (**b**) violin plot of guard cell length (m) of all species in impressions and cleared leaves, with average labeled below each violin. (**c**) violin plot of guard cell width (m) of all species in impressions and cleared leaves, with average labeled below each violin. Whisker lengths are equivalent to 1 sigma (i.e., one standard deviation from the mean, representing 68% of all the data for each measurement).

We report first lumped and then species-specific results in detail. Measured stomatal density from stomatal impressions for *Thuja* ranged from 5.28 × 10^7^ stomata m^−2^ to 5.95 × 10^8^ stomata m^−2^ (average of 2.60 × 10^8^ stomata m^−2^; Table 1; Figure 1), and for chromium-cleared *Thuja* leaves ranged from 1.06 × 10^8^ stomata m^−2^ to 4.45 × 10^8^ stomata m^−2^ (average of 2.46 × 10^8^ stomata m^−2^; Table 1; Figure 1).

**Figure 2:**
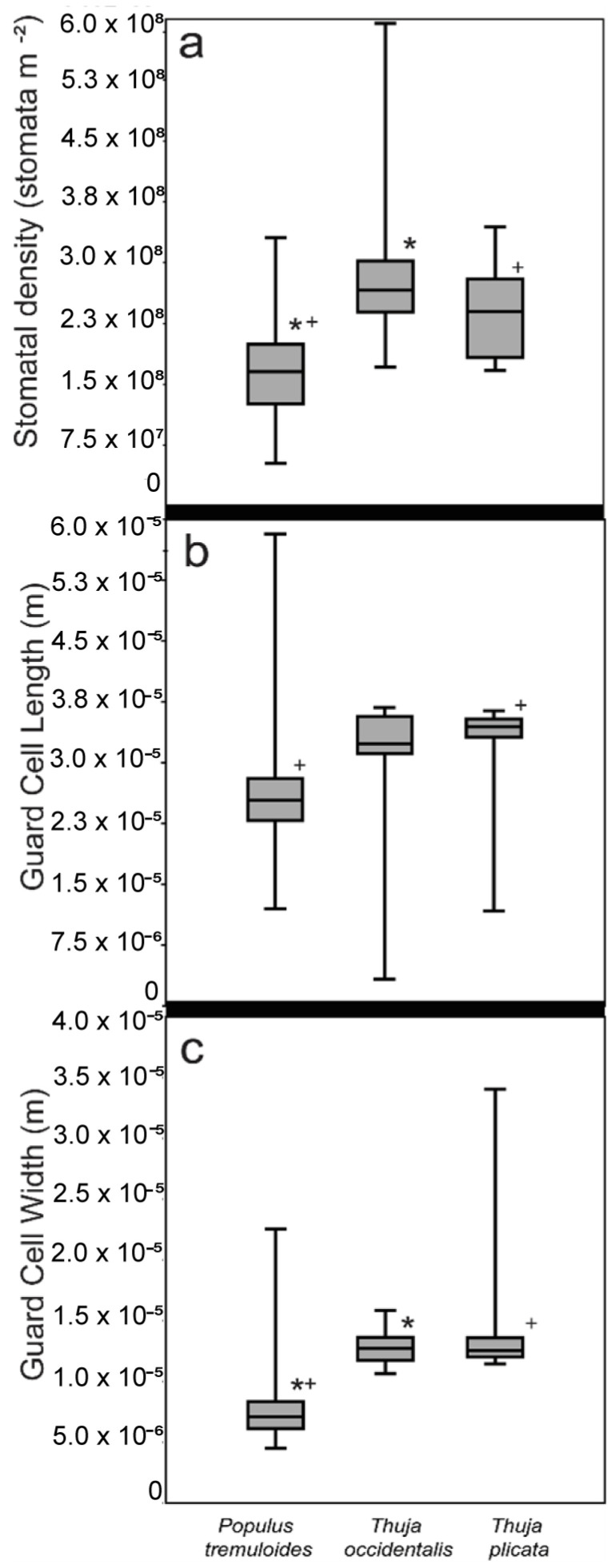
Stomatal parameter comparison of (**a**) stomatal density, (**b**) guard cell length, (**c**) guard cell width, for each species of all leaves prepared using the stomatal impression method. Asterisks (*) denote statistically significant difference between the parameters of *Populus tremuloides* and *Thuja occidentalis* (stomatal density and guard cell width) as determined by a 2-tailed *t*-test. Plus symbols (+) denote statistically significant differences between the parameters of *Populus tremuloides* and *Thuja plicata* (guard cell length and guard cell width). There were no statistically significant differences between any of the measured parameters of *Thuja occidentalis* and *Thuja plicata*.

**Figure 3:**
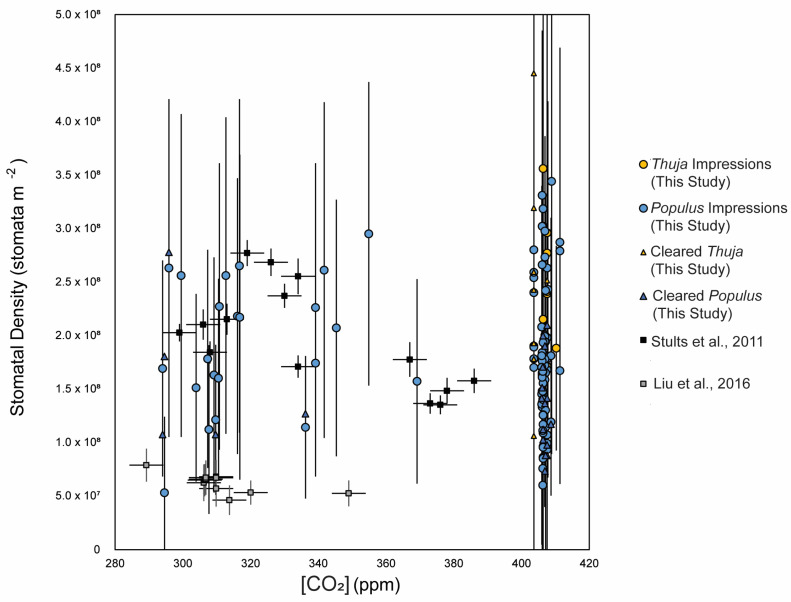
Global [CO_2_] for the sample date (by month and year) as measured at Mauna Loa Observatory after 1957 [52] and in ice cores before 1957 [53] compared with stomatal density measured on stomatal impressions and cleared leaves for Populus (blue circles for impressions, blue triangles for cleared leaves), and Thuja (yellow circles for impressions, yellow triangles for cleared leaves) and data using the same techniques as those in this study, presented by Stults et al. (2011, black squares) [54] and Liu et al. (2016, grey squares) [55]. For *Thuja*, there was a moderate correlation between stomatal density measured on stomatal impressions and chromium-cleared leaves (r = 0.49, R^2^ = 0.24; m = 0.66; *p*-value = 0.008; Figure 6a), but no correlation between GCL measured on the stomatal impressions and GCL measured on chromium-cleared leaves (r = 0.17; m = −0.16; Figure 6c). There was likewise no predictive relationship between GCW measured on impressions and chromium-cleared leaves; in fact, the slope between GCW measured on leaves treated in each way was negative (m = −0.67; Figure 6b).

**Figure 4:**
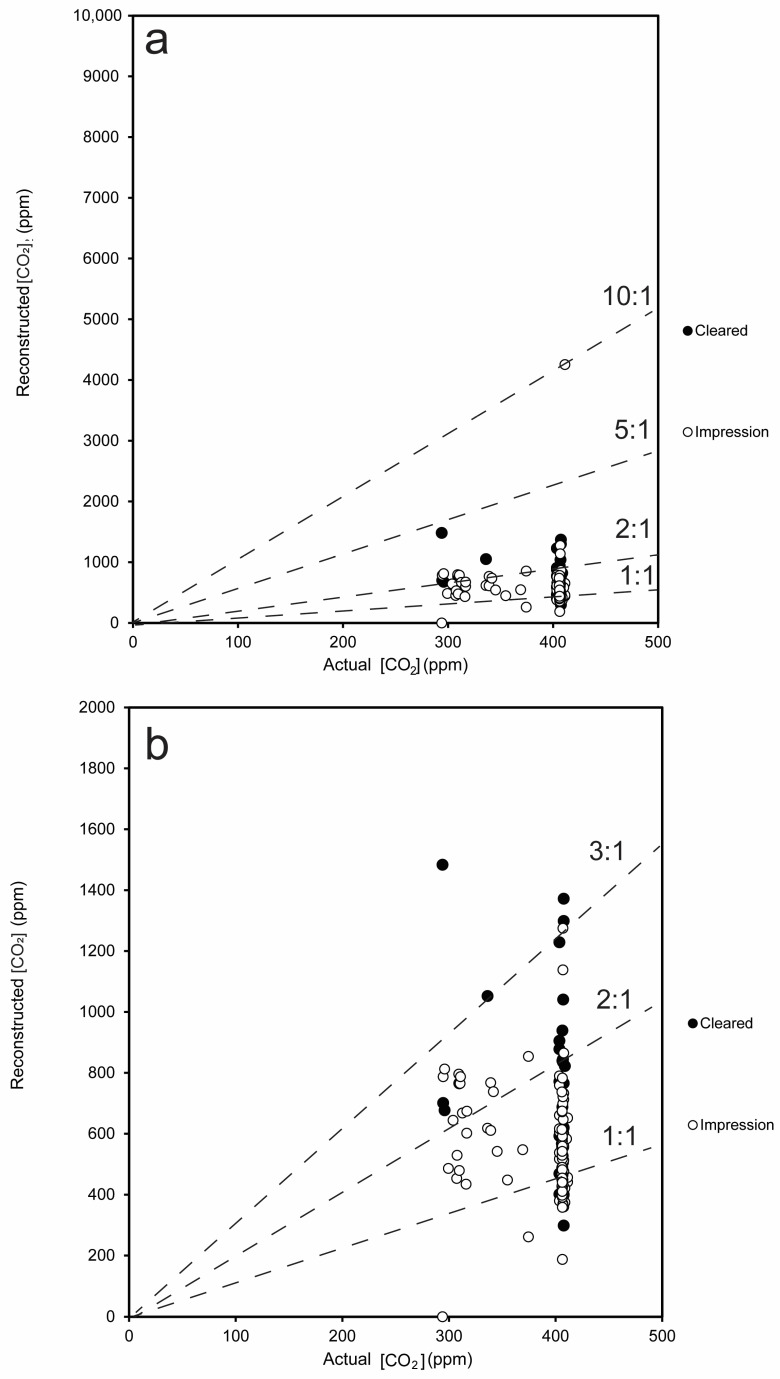
Actual CO_2_ compared with [CO_2_] reconstructed using the measurements in this study and gas-exchange model for parameters measured on cleared leaves (black circles) and stomatal impressions (white circles). Dashed lines demonstrating 10:1, 5:1, 2:1, and 1:1 relationships between actual and reconstructed values are shown in (**a**), while (**b**) shows all points excluding outliers over 1500 ppm (geologically reasonable for Cenozoic Era) values, including 3:1, 2:1, and 1:1 relationships in dashed lines.

**Figure 5:**
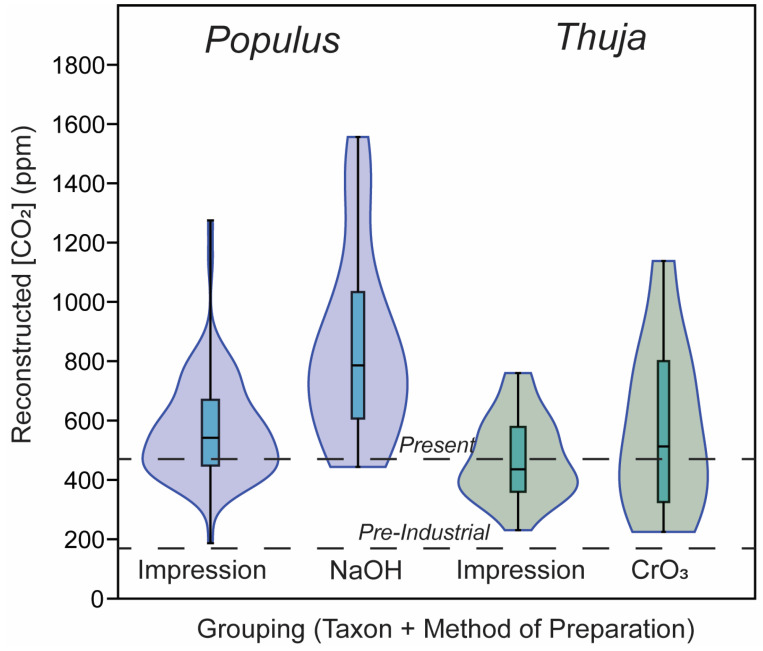
Reconstructed [CO_2_] value data distribution for impressions and cleared leaves. Dashed lines show Pre-Industrial [CO_2_] values (180 ppm) and modern [CO_2_] values (420 ppm). All values based on leave should fall between these lines.

For *Thuja*, GCL for impressions ranged from 1.17 × 10^−5^ m to 5.82 × 10^−5^ m (average of 3.17 × 10^−5^ m; Table 1; Figure 1) and chromium-cleared leaves ranged from 2.35 × 10^−5^ m to 3.53 × 10^−5^ m (average of 2.98 × 10^−5^ m; Table 1; Figure 2). GCW from impressions ranged from 4.38 × 10^−6^ m to 3.39 × 10^−5^ m (average of 1.34 × 10^−5^ m; Table 1; Figure 2) and chromium-cleared leaves ranged from 3.43 × 10^−6^ m to 1.87 × 10^−5^ m (average of 9.01 × 10^−6^ m; Figure 2, Figure 3, Figure 4 and Figure 5; Table 1).

Guard cell length, GCW, and stomatal density varied depending on the species (Figure 3). On impressions, guard cell length ranged from 1.20 × 10^−5^ m to 5.82 × 10^−5^ m for *Populus* (average = 2.58 × 10^−5^ m ± 6.07 × 10^−6^, standard deviation, n = 82). Guard cell length for *Thuja* ranged from 1.17 × 10^−5^ m to 3.68 × 10^−5^ m (average = 3.17 × 10^−5^ m ± 7.77 × 10^−6^, standard deviation, n = 24). GCW ranged from 4.38 × 10^−6^ m to 2.24 × 10^−5^ m for *Populus* (average = 7.60 × 10^−6^ m ± 2.78 × 10^−6^ m, standard deviation, n = 82), and from 1.05 × 10^−5^ m to 3.39 × 10^−5^ for *Thuja* (average = 1.34 × 10^−5^ m ± 4.50 × 10^−6^, standard deviation, n = 24). Stomatal density ranged from 5.28 × 10^7^ stomata m^−2^ to 3.31 × 10^8^ stomata m^−2^ for *Populus* (average = 1.71 × 10^8^ stomata m^−2^ ± 5.95 × 10^7^, standard deviation, n = 82), from 1.67 × 10^8^ stomata m^−2^ to 5.95 × 10^8^ stomata m^−2^ for *Thuja* (average = 2.60 × 10^8^ stomata m^−2^ ± 8.92 × 10^7^, n = 24). There was a statistically significant difference between stomatal density and GCW measured on *Populus* and *Thuja* impressions (Table 1 and Table 2; Figure 2 and Figure 7).

### 3.2. CO_2_ Reconstructions Using Both Methods

**Figure 6:**
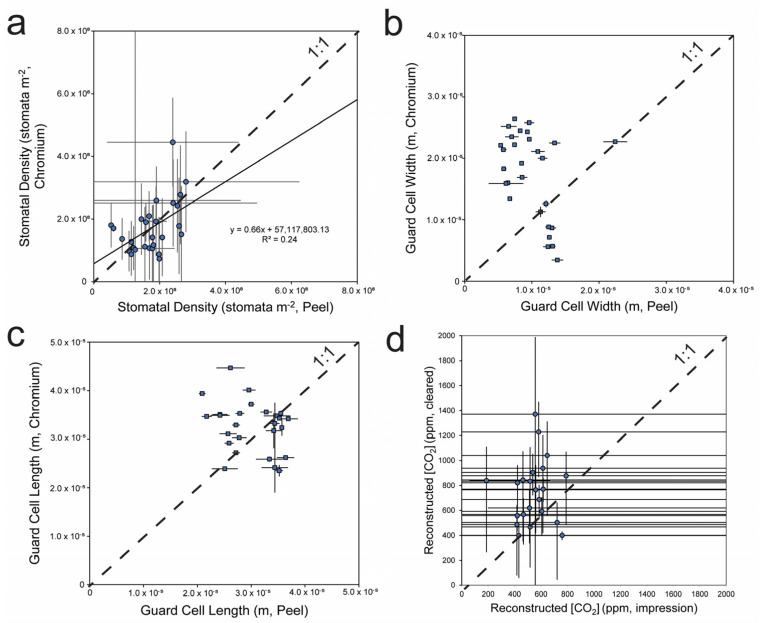
Stomatal parameter comparison for all (*Populus* and *Thuja*) leaves prepared with both chemical clearing and stomatal impressions. (**a**) Stomatal density (stomata m^−2^), (**b**) guard cell length (m), (**c**) guard cell width, and (**d**) reconstructed [CO_2_] are shown with standard errors calculated from the standard deviation of repeat measurements (s) divided by the square root of the number of measurements.

Stomatal density did not have any significant predictive relationship with [CO_2_] for any of the taxa measured *in this study* (Figure 2). Reconstructed [CO_2_] using the gas exchange model slightly exceeded actual atmospheric [CO_2_] for the impressions, with three outliers > 1500 ppm (the highest value measured throughout the Cenozoic [56]) and most other values ranging between 1 and 3× as much as actual [CO_2_] (Figure 4a,b). For all stomatal impression-based CO_2_ values below 1500 (n = 103, excluding the three outliers, Figure 4a,b and Figure 5), values ranged from 187 to 1275 ppm, with an average of 555 ppm (standard deviation = 165). For all chromium-based measurements, values ranged from 400 to 1229 ppm (average = 693, standard deviation = 293; Figure 5). For all sodium-hydroxide-based measurements, values ranged from 488 to 1483 ppm (average = 843, standard deviation = 283). For the ten *Thuja* leaves measured with both chromium and impression methodologies, reconstructed CO_2_ values from stomatal impressions ranged from 432 to 790 ppm (excluding the one value >>1500 ppm) and from cleared leaves ranged from 298 to 1229 ppm. For the fifteen *Populus* leaves measured with both cleared and impression methodologies, reconstructed CO_2_ values from stomatal impressions ranged from 187 to 721 and from cleared leaves ranged from 488 to 1372 ppm. Of these 103 samples with <1500 ppm reconstructed CO_2_, 29 of these samples fell within 50 ppm of the actual global [CO_2_] at the time (Figure 6d).

## 4. Discussion

As mentioned, we compared CrO_3_ and NaOH leaf clearing versus impression techniques on *Thuja plicata*, *Thuja occidentalis*, and *Populus tremuloides* leaf specimens. While stomatal density was comparable using the two methods (Figure 6a). These values are comparable to published values of stomatal parameters [54,55] on scale-shaped leaves (Cupressaceae and Podocarpaceae, respectively), which ranged from 1.35 × 10^8^ stomata m^−2^ to 2.77 × 10^8^ stomata m^−2^ (average = 1.98 × 10^8^ stomata m^−2^) and 4.59 × 10^7^ stomata m^−2^ to 7.86 × 10^7^ stomata m^−2^ (average 6.23 × 10^7^ stomata m^−2^), respectively (Figure 1). Each of these datasets mentioned has a substantial number of measurements (n > 10) with paired CO_2_ measurements [51,57].

GCL and GCW, two important measurements in the gas exchange model [15], were significantly different (*p* = 0.003 and *p* < 0.001, respectively, for the two-tailed *t*-test; Table 3; Figure 6b,c). When compared directly, GCL and GCW measurements either were not predictive or were negatively predictive (not a meaningful result) between cleared and impression samples (R^2^ < 0.01 and R^2^ = 0.54 with a negative correlation, respectively; Figure 6b,c). The significant differences as determined by the *t*-test (Table 3) and coefficients of determination show that one cannot use results from the impressions to predict results measured on cleared leaves. Because creating an impression requires using slight physical force to press the nitrocellulose-ethyl acetate onto the tape from the dental putty mold, if the nitrocellulose-ethyl acetate and tape are not pressed together sufficiently, the GCW will appear smaller. GCW is the finest scale measurement for the stomatal parameters input into the gas exchange model and, thus, the most sensitive to human error or shrinking dental putty.

**Figure 7:**
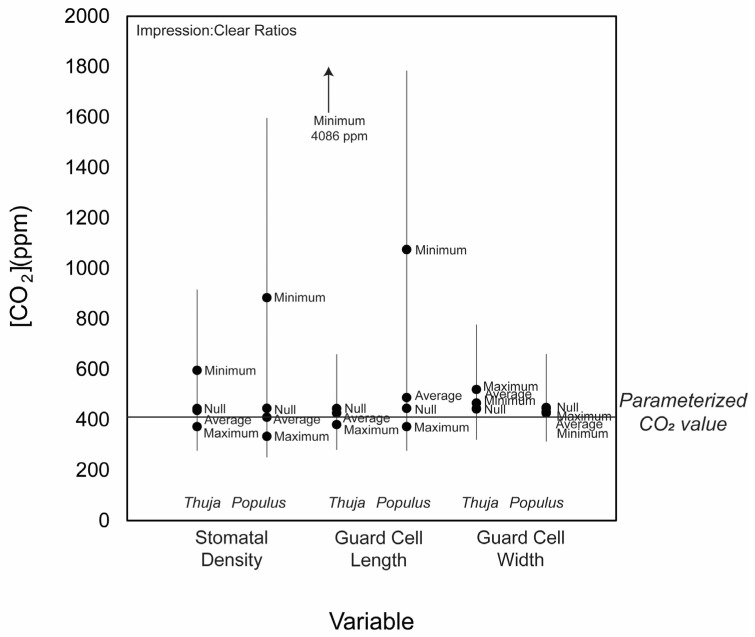
Simulations of what changing parameters involved in the gas-exchange model (guard cell width, stomatal density, and guard cell length) do, according to measured impression and cleared leaf stomata. For each parameter, “Null” demonstrates an unaltered test case, as provided by Franks et al. (2014) supplemental text [13]. Each parameter also includes this unaltered case multiplied by the average ratio of impression: cleared leaf measurement (“Average Impression:Clear”), the maximum ratio of impression: cleared leaf measurement (“Max. Impression:Clear”), and the minimum ratio of impression: cleared leaf measurement (“Min. Impression:Clear”). For example, the minimum ratio of stomatal density on *Thuja* impressions as compared with chromium-cleared leaves is 0.54, thus the test-case stomatal density for *Thuja* was multiplied by 0.54, and for *Populus*, 0.29, and run through the model. The average ratio of stomata density on the *Thuja* stomatal impression as compared with the chromium-cleared leaf is 1.03, so the average impression: clear for stomatal density represents the model results when the stomatal density is multiplied by 1.03. For *Populus*, this ratio is 1.26.

We did not use the chromium-based methodology on the angiosperm group because this highly corrosive solution causes broad-leaved leaves like angiosperms to dissolve quickly [58], while tougher, scale-like leaves of *Thuja* require corrosive solutions [59,60]. We cleared *Populus* leaves using sodium hydroxide, a more common technique for angiosperms [43]. Measurements made using both clearing and impression methodologies reconstructed reasonable, if slightly high (<3×) CO_2_ values compared with known measured CO_2_ values at sampling time (Figure 7). Importantly, stomatal density measured on leaves cleared using CrO_3_ and dental putty impressions were comparable. This indicates that in cases where dental putty impressions are the only available options (due to fragile cuticles or other complications), CO_2_ estimations based on stomatal density and/or stomatal index measurements introduce much less error than those incorporating pore and guard cell length and width measurements. The slope of the relationship between stomatal density measurement made on impressions and clearing methodology was close to 0.66 and had an intercept (i.e., discrepancy) within the standard deviation of all samples (5.7 × 10^7^ stomata m^−2^; Figure 6a), with a coefficient of determination (R^2^) of 0.24.

The impression methodology is as accurate as the standard, corrosive methodology for stomatal density measurements. However, the impression methodology is not as accurate as clearing for any estimates that require nuanced measurements on the micrometer scale (i.e., GCW, GCL), possibly reflecting shrinkage in dried versus hydrated leaves. This includes gas-exchange models. Thus, for proxy reconstructions based on gas-exchange models [13], the more precise methodology of leaf clearing is encouraged.

While this creates limitations for the use of impressions for complex plant morphology-based models, there are logistical circumstances in which the impression-based methodology is more useful. Not only is the impression methodology as accurate as typically cleared leaves, but because these impressions were non-toxic and non-destructive, they contributed significantly less waste in the laboratory than using CrO_3_ or NaOH. This methodology is most appropriate for classroom settings, where it is quicker and safer than CrO_3_ and thus accessible to a wide array of learners.

## 5. Conclusions

Both impression-based and cleared-leaf-based measurements provided viable [CO_2_] estimates using the gas exchange model for measurements made on historical and modern leaves of both types of leaves evaluated: those of scale-leaf conifers (*Thuja*) and of broad-leaf angiosperms (*Populus*). Impression-based measurements were less accurate for GCW and GCL because of shrinkage due to drying and the imprecision of the physical mechanisms involved in making the impression (i.e., human error), but stomatal density measurements were comparable and correlated between the two methods. Furthermore, the parameters measured between broad-leaf angiosperms and scale-leaf conifers are different, demonstrating the importance of taxonomically diverse starting parameters when using the gas-exchange model to reconstruct paleo-CO_2_. However, the variability of parameters within the *Thuja* genus was not significant. Future work evaluating multiple closely related species from the same genus is needed to evaluate the extent to which subscribing to *one* equation for genera, such as one *Thuja* equation, is appropriate.

Based on these findings, we recommend that leaf clearing methodology (CrO_3_, NaOH) be employed as much as possible when using a gas exchange model, but if only stomatal density or index are available due to logistical parameters, either approach is potentially viable. Further work covering a wider time slice of Industrialization with a diversity of plant functional types can demonstrate the efficacy of this tool using the stomatal density and gas exchange models. Additionally, future work should include development on the methodology of creating impressions of stomata from fossils, as this is harder than in modern material.

## Figures and Tables

**Table 1:** The number of samples, average, and standard deviations of each parameter, measured for each species, divided by preparation method.

	n	Average Stomata Density (Stomata m^−2^)	Standard Deviation Stomata Density (Stomata m^−2^)	Average Guard Cell Length (m)	Guard Cell Length Standard Deviation (m)	Average Guard Cell Width (m)	Guard Cell Width Standard Deviation (m)
*Populus tremuloides* impressions	82	1.71 × 10^8^	5.95 × 10^7^	2.58 × 10^−5^	6.07 × 10^−6^	7.56 × 10^−6^	2.78 × 10^−6^
*Thuja* impressions	24	2.60 × 10^8^	8.92 × 10^7^	3.17 × 10^−5^	7.77 × 10^−6^	1.34 × 10^−5^	4.50 × 10^−6^
**All impressions**	**106**	**1.91 × 10^8^**	**7.68 × 10^7^**	**2.71 × 10^−5^**	**6.91 × 10^−6^**	**8.92 × 10^−6^**	**4.05 × 10^−6^**
CrO_3_-cleared *Thuja*	10	2.46 × 10^8^	9.67 × 10^7^	2.98 × 10^−5^	4.35 × 10^−6^	9.01 × 10^−6^	4.36 × 10^−6^
NaOH-cleared *Populus*	20	1.41 × 10^8^	5.10 × 10^7^	3.55 × 10^−5^	7.04 × 10^−6^	2.12 × 10^−5^	3.61 × 10^−6^
**All cleared leaves**	**30**	**1.76 × 10^8^**	**8.46 × 10^7^**	**3.36 × 10^−5^**	**6.76 × 10^−6^**	**1.72 × 10^−5^**	**6.98 × 10^−6^**

**Table 2:** *t*-test *p*-values reported for comparisons of each species for stomatal density, guard cell length, and guard cell width in impressions (selected because of the higher number of samples). *p*-values < 0.01 are highlighted in green and bolded, demonstrating significant differences in mean values for the compared species.

Stomatal Density	*Populus tremuloides*	*Thuja occidentalis*
*Populus tremuloides*		
*Thuja occidentalis*	**<0.01**	
*Thuja plicata*	**<0.01**	0.13
**Guard Cell Length**	*Populus tremuloides*	*Thuja occidentalis*
*Populus tremuloides*		
*Thuja occidentalis*	0.13	
*Thuja plicata*	**<0.01**	0.56
**Guard Cell Width**	*Populus tremuloides*	*Thuja occidentalis*
*Populus tremuloides*		
*Thuja occidentalis*	**<0.01**	
*Thuja plicata*	**<0.01**	0.41

**Table 3:** Mean values reported for comparison measurements on cleared leaves and impressions, and the *p*-value for the two-tailed *t*-test comparing the means of both.

Value	Stomatal Density (Stomata m^−2^)	Guard Cell Length (m)	Guard Cell Width (m)
Mean for impressions	1.74 × 10 ^8^	2.97 × 10^−5^	9.95 × 10^−6^
Mean for cleared leaves	1.70 × 10^8^	3.37 × 10^−5^	1.72 × 10^−5^
*p*-value	0.83	0.05	<0.001

## Data Availability

Data available in a publicly accessible repository. The data presented in this study are openly available in Mendeley Data Repository at [doi: 10.17632/gs6rn9tjxn.1; accessed on 13 November 2023], reference number [61].

## Supplementary Materials

The following supporting information can be downloaded at: https://www.mdpi.com/article/10.3390/life14010078/s1, Supplemental Text; Figure S1: All Environmental Parameters versus Stomatal Parameters; Supplemental Data Tables: All Measurements—Data are available at Mendeley Data: 10.17632/gs6rn9tjxn.1 (accessed on 13 November 2023).
