# Supplementary material for: Comparing Methodologies for Stomatal Analyses in the Context of Elevated Modern CO2
Source: Life (Basel). 2024 Jan 2;14(1):78. doi: 10.3390/life14010078 (PMC10821100; doi:10.3390/life14010078)

Supplemental Material

1. Supplemental Results Text
2. Supplemental Figure
3. Supplemental Data Tables – Data are available at Mendeley Data: 10.17632/gs6rn9tjxn.1

1.Supplemental Results

Individual stomatal parameters and environmental variables

While [CO_2] is the most significant control on stomatal parameters, we note modest relationships between these parameters and other spatial environmental factors that account for some of the variance between modeled and measured [CO_2]. For example, stomatal density is related to elevation, GCL to precipitation, and GCW to both precipitation and elevation (Figure S1c,e,h,i), each of these environmental parameters only predicted 0 to 12% of change in stomatal parameters (R^2 range from 0.00 to 0.12; Figure S1). There is a slight correlation between parameters and elevation (which is in turn, inversely related to CO_2), and a relationship between several parameters and mean annual precipitation due to the key role that stomata play in photosynthesis as the major influx point for CO_2 *and* export point for water. There is a modest negative correlation between stomatal density and elevation (r = -0.32; Figure S1c). The relationship between elevation and GCW is modest and inverse as well (r = -0.33; Figure S1i), related to overall leaf size, which decreases across many species with increased elevation (Midolo et al. 2019). As elevation increases, temperature decreases and irradiance increases, thus leaf area decreases (Porter et al. 2009; Wright et al. 2017). There is likewise a weak correlation between precipitation and guard cell length and width (Figure S1e,h), however, all of the samples taken above 2000 mm yr^{-1} (which largely drive the trend) are members of the *Thuja*

genus, thus, this could be related to individual species niches and to what plants thrive at higher precipitation regimes. Previous works by Stein et al., (2021a) and others (Sheldon et al. 2020; Hare & Lavergne 2021) have found that trends in carbon isotope ecology metanalyses likewise disappear on a plant functional-specific and/or species-specific level, including for precipitation (Stein et al., 2021b). Thus, this work affirms that plant response to the environment is deep-seated in evolutionary adaptations and is not ubiquitous even among species from overlapping ranges.

Species-specific Findings

The use of stomatal density as a proxy for paleo-[CO_2] has been most commonly applied to *Ginkgo* leaves and to broad-leaf angiosperms (Beerling & Royer 2002). Recent studies have identified inter-species parameter variability, including within conifers (Porter et al., 2019; Liang et al., 2022), that suggest a broad array of training data are needed rather than "universal" calibrations. Gymnosperms are overall less responsive to changes in water availability in the environment, due to the necessary high water use efficiency related to elevated temperatures during their origin (Klein & Ramon 2019; Hare & Lavergne 2021; Yang et al. 2021). As such, gymnosperms tend to have larger stomata (e.g., Franks & Beerling 2009; McElwain et al. 2016a, Xiong et al. 2018; Xiong & Flexas 2020).

Yet, these scale-like leaves are scarce in proxy calibrations and often abundant in the fossil record (Haworth et al., 2010). We focused on any variability in parameter measurements across angiosperms and gymnosperms using both of the different measurement methods. In our measurements, we saw the stomata density measured in scale leaves significantly exceeded those for *Populus* by 1.5x (average of 1.71×10^8 compared to 2.60×10^8, Figure 2a. GCL for is significantly larger (see Figure 2b) than length measured on *Populus.* GCW for *Thuja* is

significantly larger (see Figure 2c) than width measured on *Populus tremuloides,* congruent with our understanding of stomata size.

References

1. Midolo, G., De Frenne, P., Hölzel, N., & Wellstein, C. (2019). Global patterns of intraspecific leaf trait responses to elevation. *Global change biology*, *25*(7), 2485-2498.

2. Poorter, H., Niinemets, Ü., Poorter, L., Wright, I. J., & Villar, R. (2009). Causes and consequences of variation in leaf mass per area (LMA): a meta-analysis. *New phytologist*, *182*(3), 565-588.

3. Wright, I. J., Dong, N., Maire, V., Prentice, I. C., Westoby, M., Díaz, S., Gallagher, R. V., Jacobs, B. F., Kooyman, R., Leishman, M. R., & Wilf, P. (2017). Global climatic drivers of leaf size. *Science*, *357*(6354), 917-921.

4. Hare, V. J., & Lavergne, A. (2021). Differences in carbon isotope discrimination between angiosperm and gymnosperm woody plants, and their geological significance. *Geochimica et Cosmochimica Acta*.

5. Stein, R. A., Sheldon, N. D., & Smith, S. Y. (2021)b. Soil carbon isotope values and paleoprecipitation reconstruction. *Paleoceanography and Paleoclimatology*, *36*(4), e2020PA004158.

6. Porter, A. S., Gerald, C. E. F., Yiotis, C., Montanez, I. P., & McElwain, J. C. (2019). Testing the accuracy of new paleoatmospheric CO2 proxies based on plant stable carbon isotopic composition and stomatal traits in a range of simulated paleoatmospheric O2: CO2 ratios. *Geochimica et Cosmochimica Acta*, *259*, 69-90.

7. Liang, J. Q., Leng, Q., Höfig, D. F., Niu, G., Wang, L., Royer, D. L., Burke, K., Xiao, L., Zhang, Y. G., & Yang, H. (2022). Constraining conifer physiological parameters in leaf gas-exchange models for ancient CO2 reconstruction. *Global and Planetary Change*, *209*, 103737.

8. Klein, T., & Ramon, U. (2019). Stomatal sensitivity to CO_2 diverges between angiosperm and gymnosperm tree species. *Functional Ecology, 33*(8), 1411-1424.

9. Yang, Y. J., Bi, M. H., Nie, Z. F., Jiang, H., Liu, X. D., Fang, X. W., & Brodribb, T. J. (2021). Evolution of stomatal closure to optimise water use efficiency in response to dehydration in ferns and seed plants. *New Phytologist*.

10. Franks, P. J., & Beerling, D. J. (2009). Maximum leaf conductance driven by CO_2 effects on stomatal size and density over geologic time. *Proceedings of the National Academy of Sciences*, *106*(25), 10343-10347.

11. McElwain, J. C., Yiotis, C., & Lawson, T. (2016). Using modern plant trait relationships between observed and theoretical maximum stomatal conductance and vein density to examine patterns of plant macroevolution. *New Phytologist, 209*(1), 94-103.

12. Xiong, D., Douthe, C., & Flexas, J. (2018). Differential coordination of stomatal conductance, mesophyll conductance, and leaf hydraulic conductance in response to changing light across species. *Plant, cell & environment*, *41*(2), 436-450.

13. Xiong, D., & Flexas, J. (2020). From one side to two sides: the effects of stomatal distribution on photosynthesis. *New Phytologist*, *228*(6), 1754-1766.

2. Supplemental Figures

Figure S1 Environmental parameters mean annual temperature (a,d,g, in grey circles), mean annual precipitation (b,e,h, in red circles) and elevation (c,f,i, in blue circles) compared to stomatal density (a-c), guard cell length (d-f) and guard cell width (g-i) in peels. Trendlines are shown in solid black lines, with equations and R^2 values in the upper right corner of each panel.

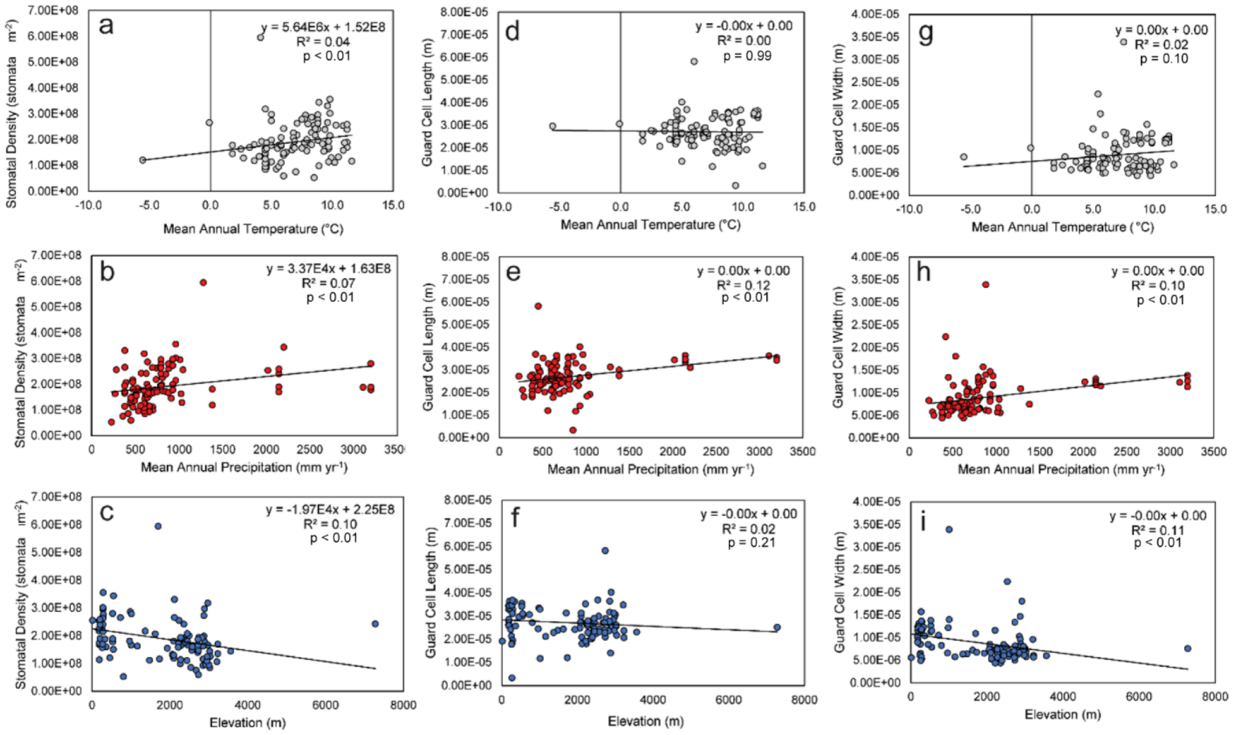

Supplement: Supplementary file 1 [file life-14-00078-s001.zip › life-2757650-supplementary.pdf]
